# PD16 Multicriteria Model For The Inclusion Of Oncologic Drugs In The Essential Medicines List: Insights From The Ecuadorian Experience

**DOI:** 10.1017/S0266462325101517

**Published:** 2025-12-29

**Authors:** Victor Flores, Paulina Quirola, Fernanda Parreño, Elizabeth Guambo

## Abstract

**Introduction:**

Including drugs in the National Essential Medicines List is a complex process that considers population, budget, and socioeconomic factors. In Ecuador, these decisions rely on efficacy and safety data from existing studies without estimating the real impact and clinical benefit of the drugs. This study proposed a multicriteria model using minimal clinically important difference (MCID) values to evaluate oncologic drugs for inclusion.

**Methods:**

Safety and efficacy were assessed using MCID values for primary outcomes, incorporating measures such as overall survival (OS), disease-free survival (DFS), and complete remission, following the Magnitude of Clinical Benefit Scale (v1.1) of the European Society for Medical Oncology. Epidemiological and demographic data were obtained from the National Institute of Statistics and quality of life was evaluated using EuroQol-5D scores. Information on oncologic drugs and regulatory considerations was sourced from authoritative organizations. All parameters were systematically organized and weighted, ensuring the total criteria groups accounted for 100 percent.

**Results:**

Seven criteria groups were established, each containing parameters evaluated on a scale from zero to five, designed to reward or penalize based on assessment goals. The groups were weighted from five to 30 percent to reflect their relative importance or impact on decision-making. The criteria and weights were as follows: (i) disease burden (30%); (ii) efficacy (30%); (iii) safety (15%); (iv) quality of life impact (5%); (v) availability and accessibility (5%); (vi) therapeutic alternatives and international recommendations (10%); and (vii) other important criteria (5%). Consequently, scores were weighted differently across groups.

**Conclusions:**

Accurately estimating the impact of oncologic drugs is critically important for public health. Implementing this multicriteria model will provide a more efficient, objective, and systematic framework for evaluating the inclusion, modification, or exclusion of oncologic drugs with respect to the National Essential Medicines List. This approach enhances decision-making processes and ensures the optimal selection of anti-cancer therapies, ultimately improving patient outcomes and resource allocation in Ecuador.

